# HIF1α or mitophagy: which drives cardiomyocyte differentiation?

**DOI:** 10.15698/cst2020.05.219

**Published:** 2020-05-11

**Authors:** Beatriz Villarejo-Zori, Juan Ignacio Jiménez-Loygorri, Patricia Boya

**Affiliations:** 1 Department of Cellular and Molecular Biology, Centro de Investigaciones Biológicas Margarita Salas, CSIC, Madrid, Spain.; # These authors contributed equally.

**Keywords:** mitophagy, HIFα, NIX, cardiomyocyte, differentiation, glycolisis

Cardiomyocytes (CMs) are the main cellular component of the heart's atria and ventricles, and their contractile capacity is the engine that drives blood flow throughout the body. The differentiation of cardiac myoblasts into CMs during heart development is an energy-demanding process, which is sustained by a metabolic shift towards glycolysis [1]. Subsequently, differentiated CMs rely almost exclusively on mitochondria-driven oxidative phosphorylation for energy production [2]. Metabolic shifts also occur during adult CM proliferation in the regenerating heart [3]. In other contexts, such as neuronal differentiation or immune cell activation, cell differentiation is sustained by the upregulation of glycolysis. In these two cell types -neurons and macrophages-mitophagy is the key regulator of the metabolic shift that is both necessary and sufficient for cell differentiation [4].

Mitophagy is a selective form of autophagy in which mitochondria are degraded inside lysosomes. In addition to its role as a quality-control mechanism, mitophagy-mediated mitochondrial degradation drives remodelling of the mitochondrial network, ensuring that the energetic requirements of each cell type are fulfilled. Mitophagy can occur via PINK1/Parkin signalling or via the mitophagy receptors. The latter pathway involves proteins (including BNIP3, NIX/BNIP3L, and FUNDC1) bearing a motif that is recognized directly by the LC3-containing autophagosomal membrane. While PINK1/Parkin-dependent mitophagy is mainly triggered by dysregulation of the mitochondrial membrane potential, receptor-mediated mitophagy can be induced by a variety of stimuli, including oxygen deficit during hypoxia and developmental signalling [5]. Interestingly, HIF1α, the master transcription factor of the cellular response to hypoxia, regulates the expression of many genes, including most regulators of glycolysis and several mitophagy receptors (e.g. BNIP3 and NIX) [6].

**Figure 1:**
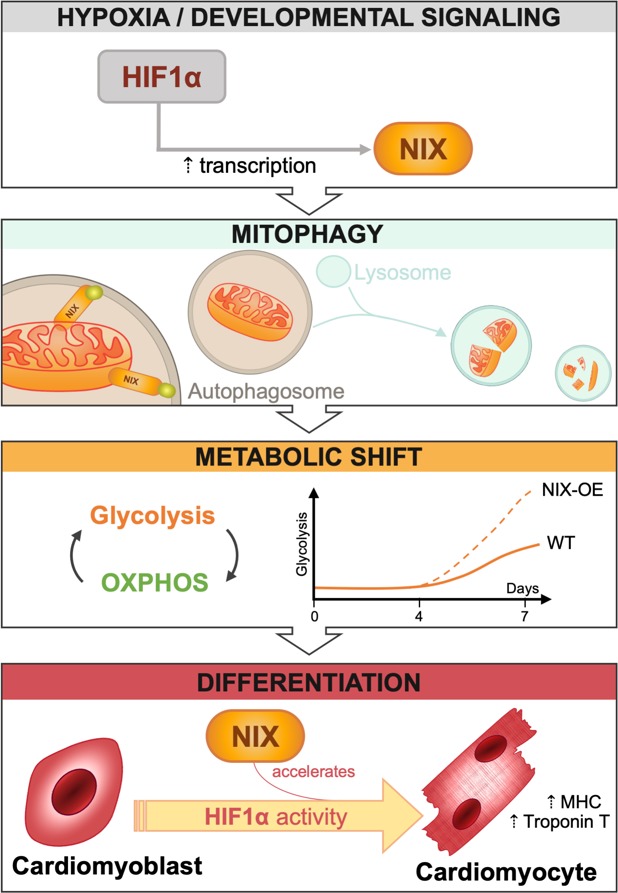
FIGURE 1: NIX accelerates cardiomyocyte differentiation. Upregulation of HIF1α activity in response to a variety of stimuli, including hypoxia and developmental signalling, modulates the bioenergetic status of the cell by triggering receptor-mediated mitophagy. These changes allow for the increase in cellular glycolytic capacity necessary to provide energy to drive cardiomyocyte differentiation. Zhao et al. demonstrate that this process is dependent on HIF1α but not on NIX. However, NIX overexpression accelerates the glycolytic shift and leads to early differentiation as evidenced by increased levels of the cardiomyocyte markers MHC and troponin T.

During heart development, receptor-mediated mitophagy is essential for correct mitochondrial network formation, although the specific details of how mitophagy impacts overall differentiation remain unclear [7]. In this issue of *Cell Stress*, Zhao and collaborators tackle this problem in H9c2 cells [8], a widely used *in vitro* model of CM differentiation that can be genetically manipulated to search for molecular regulators of CM development. When cultured for 7 days in the presence of low serum and retinoic acid, these cells express troponin and other CM markers such as MHC. Over this 7-day period of CM differentiation, the authors demonstrate a progressive increase in mRNA and protein levels of HIF1α, BNIP3, and hexokinase 2 (HK2), as well as concomitant increases in the expression of differentiation markers such as troponin T and MHC, suggesting a link between hypoxia, mitophagy receptor expression, and cell differentiation. Mitophagy also increased as CMs differentiated, and was dependent on HIF1α levels. Importantly, silencing of HIF1α on day 4 decreased mitophagy and troponin T expression. Confirming that this phenotype is the result of HIF1α transcriptional activity, expression of a constitutively stable HIF1α mutant led to increased HIF1α-dependent transcription and mitophagy. Taken together, these data indicate that HIF1α induces expression of the mitophagy regulator NIX, increases mitophagy, and promotes CM differentiation **(Fig. 1)**.

To decipher the role of NIX in the CM differentiation process, the authors silenced NIX and BNIP3 expression on day 4 of the differentiation process using corresponding siRNAs. The results clearly demonstrate that downregulation of NIX, but not BNIP3, reduces mitophagy. However, although NIX is the main mitophagy regulator in H9c2 cells, its downregulation does not reduce the expression of markers of cell differentiation. Thus, while HIF1α appears essential to regulate CM differentiation, NIX-dependent mitophagy seems to be dispensable.

Several hypotheses can be put forth to explain these intriguing findings. First, it has been reported in the literature that the expression of some mitophagy receptors (NIX, BNIP3, and FUNDC1) may increase in the absence of others, although doubts remain as to whether such compensation occurs at the functional level [7, 9]. Downregulation of more than one mitophagy receptor may be necessary to completely blunt mitophagy in some cell types [5, 10]. Zhao *et al.* did not observe this compensatory relationship between NIX and BNIP3 in their experimental conditions: MHC and troponin expression remained normal even when both NIX and BNIP3 were silenced. Second, as stated by the authors, the process of HIF1α-dependent CM differentiation may have already begun before day 4. The authors chose to downregulate NIX on day 4 as this is the time point at which both HIF1α expression and mitophagy begin to increase. This important question could be investigated using NIX-knockout H9c2 cells. Third, other as-yet-undiscovered mitophagy regulators may participate in CM differentiation.

While NIX downregulation did not reduce the expression of the aforementioned differentiation markers, its overexpression did increase mitophagy and the expression of the differentiation markers troponin T and MHC. Moreover, this effect was specific to NIX; BNIP3 overexpression had no effect on mitophagy or on cell differentiation. What then is the role of NIX in the differentiation process? Does NIX-dependent mitophagy play a metabolic role in facilitating differentiation, as occurs during neuronal differentiation? Some of the authors' findings indeed support this view. NIX overexpression increased mitophagy by almost 2-fold; reduced the oxygen consumption rate (OCR), suggesting reduced mitochondrial activity; and increased protein levels of HK2 and PKM2, both of which are key glycolysis regulators. These observations suggest that increased NIX expression may aid the removal of mitochondria, promoting a shift toward glycolysis that facilitates or favours cell differentiation.

The authors also report that mitochondrial biogenesis increased during CM differentiation. After 7 days *in vitro*, they observed increases in levels of PGC1a, the master regulator of mitochondrial biogenesis, and in the activity of the mitochondrial enzyme citrate synthase. In line with these findings, mitochondrial activity, as determined by measuring the OCR, was higher on day 7 than day 0. This begs the question: why do both mitophagy and mitochondrial biogenesis increase during the CM differentiation process? One possible explanation is that the two processes are not mutually exclusive. Palikaras *et al.* showed that in *Caenorhabditis elegans* a coordinated interface between mitochondrial biogenesis and mitophagy promotes cell survival during aging and stress resistance [11]. Interestingly, this effect is mediated by SKN-1, the nematode homolog of mammalian nuclear factor erythroid-derived 2-like 2 (Nrf2/NFE2L2), which regulates the expression of DCT-1, the *C. elegans* homolog of BNIP3 and BNIP3L/NIX, and other mitochondrial genes. In addition, simultaneous increases in both mitophagy and biogenesis during heart maturation could underlie changes in mitochondrial type. Mitochondrial type changes during heart development, since adult hearts mainly oxidize fatty acids in the mitochondria, while foetal cardiomyocytes preferentially metabolize carbohydrates. Thus, the selective elimination of foetal mitochondria together with the proliferation of more mature mitochondria results in a rapid change in the mitochondrial pool during heart maturation [12].

The findings of this study raise yet another question: how can NIX overexpression and HIF1α downregulation, which exert opposing effects on cell differentiation, both reduce OCR? One could speculate that the decrease in mitophagy resulting from HIF1α downregulation could lead to the accumulation of damaged (and hence less active) mitochondria, in turn resulting in a decrease in OCR. Maintaining a pool of healthy mitochondria is particularly important in tissues with a high energy consumption rate, such as cardiac muscle [13].

In conclusion, this interesting study demonstrates that HIF1α is required for CM differentiation. Mitophagy, on the other hand, appears to be dispensable, but may nonetheless participate, accelerating the rate of cell differentiation. The data presented suggest that the requirement of mitophagy for cell differentiation is cell type-dependent. Supporting this view, our research group has shown that NIX-dependent mitophagy is an absolute requirement for retinal ganglion cell (RGC) differentiation [4]. Specifically, NIX-dependent mitophagy is essential to induce a metabolic shift towards glycolysis, and RGC number is reduced in NIX-deficient animals. Interestingly, during neurogenesis, RGCs must generate a very long axon that travels from the eye to synapse with target neurons located millimetres away in the brain. The massive anabolic requirements of these cells are fulfilled by glycolysis. Once the RGC axon reaches its target, cell differentiation is completed and metabolism shifts to rely on oxidative phosphorylation as an energy source.

Zhao *et al.* have demonstrated the importance of hypoxia-mediated mitophagy in cardiomyocytes, and have shed light on the potential role of NIX-dependent mitophagy as an accelerator of cell differentiation. Hypoxia regulates mitophagy, mitochondrial biogenesis, metabolism, and cell differentiation, but while HIF1α is necessary NIX-dependent mitophagy is not. The authors also show that mitophagy is increased in the prenatal heart, peaking at E17.5. Crucially, levels of mitophagy are not uniform in all regions of the developing heart, suggesting the existence of different “niches”, which may reflect groups of cells at different stages of differentiation and with distinct metabolic requirements. Mitophagy has been recently shown to exert protective effects in several cardiovascular conditions, including myocardial infarction and ischemia-reperfusion injury [14–18], and thus appears to play a key role in cardiac physiology. Future studies will help to further elucidate the consequences of mitophagy-mediated control of mitochondrial number and type, and of the cell's metabolic status during heart development.
